# Resolving a paradox about how vision is transformed into familiarity

**DOI:** 10.1101/2025.06.13.659490

**Published:** 2025-06-14

**Authors:** Simon Bohn, Catrina M. Hacker, Barnes G. L. Jannuzi, Travis Meyer, Madison L. Hay, Nicole C. Rust

**Affiliations:** Department of Psychology, University of Pennsylvania, Philadelphia, USA

## Abstract

While humans and other primates are generally quite good at remembering the images they have seen, they systematically remember some images better than others. Here, we leverage the behavioral signature of “image memorability” to resolve a puzzle around how the brain transforms seeing into familiarity. Namely, the neural signal driving familiarity reports is thought to be repetition suppression, a reduction in the vigor of the population response in brain regions including inferotemporal cortex (ITC). However, within ITC, more memorable images evoke higher firing rate responses than less memorable ones, even when they are repeated. These two observations appear to conflict: if *reduced* firing leads to stronger memory signaling, then why are the images that induce *greater* firing more memorable? To resolve this paradox, we compared neural activity in ITC and the hippocampus (HC) as two rhesus monkeys performed a single-exposure image familiarity task. We found evidence that the paradox is resolved in HC where neural representations reflected an isolated memory signal that was larger for more memorable images, but HC responses were otherwise uncorrupted by memorability. Memorability behavior could not be accounted for by trivial computations applied to ITC (like thresholding). However, it could be decoded from ITC with a linear decoder that corrects for memorability modulation, consistent with the hypothesis that ITC reflects familiarity signals that are selectively extracted through medial temporal lobe (MTL) computation. These results suggest a novel role for the MTL in familiarity behavior and shed new light on how the brain supports familiarity more generally.

## Introduction:

Understanding how the brain stores information about experiences and remembers that information later is central to understanding how memory works. In the case of visual familiarity (“Have you seen this before?”, also called “recognition memory”), one well-established behavioral signature is the manner in which images are systematically more memorable or forgettable, a property called “image memorability”. Image memorability is shared across human observers^1–6^ and is correlated between humans and rhesus monkeys^7^. Likewise, image memorability is reflected in deep artificial neural networks that are not explicitly trained for it but rather to categorize object identity^7,8^, suggesting that it is a natural byproduct of a system wired up to see. Here we use image memorability variation as a lever to pinpoint how the brain transforms seeing into remembering.

**Figure 1:**
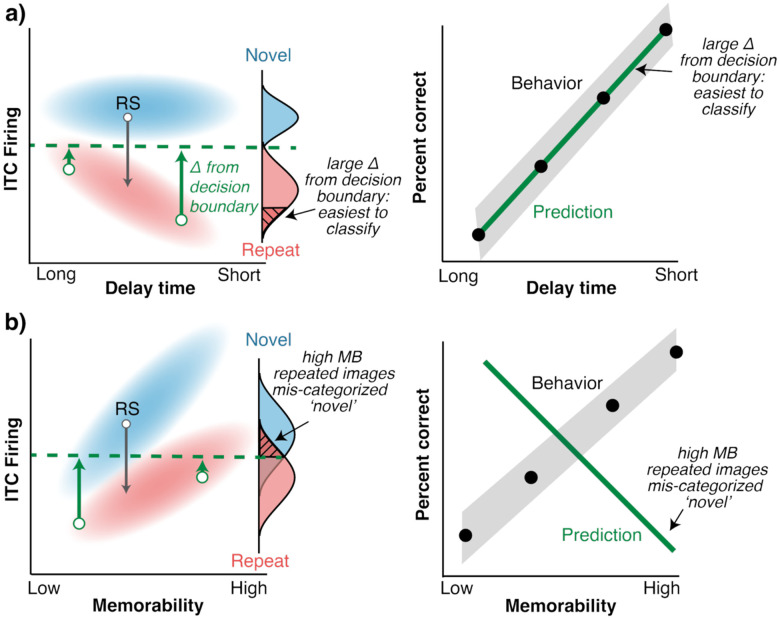
The familiarity-memorability paradox. Left-side panels reflect hypothetical population grand mean firing rates; blue and red clouds reflect distributions of firing rates to novel and repeated images, respectively. Repetition suppression is the difference between the novel and repeated firing rate distributions, indicated with a solid gray arrow, and the implementation of a simple “spike count” classifier is shown by drawing a decision boundary at the overall mean firing rate (from the perspective of a downstream, brain area, the only information available is the firing rate). The distance from two exemplar points to the decision boundary is shown (left, green arrows). Projections of the firing rate clouds onto the classifier’s axis are shown as approximate probability density functions. Reading out this classifier would classify any image (regardless of the ground truth) above the boundary as novel, and anything below it as repeated. **a)** The repetition suppression hypothesis proposes that reductions in overall population vigor drive reports of familiarity, where repetition suppression is strongest immediately after seeing an image and disappears as a function of delay time. In this case, a simple linear decoder of ITC firing rate (right, solid green) predicts forgetting behavior (right, black dots; idealized here for conceptual simplicity). **b)** The paradox occurs when ITC population response vigor is also modulated by the images themselves, for instance, by memorability, where more memorable images produce higher ITC firing rates (left). In this case, an ITC firing rate decoder predicts that the most memorable images (solid black arrows, pointing to high memorability (MB) images) are the least memorable (because they have the highest firing rates), at odds with behavior (right, solid black dots).

Considering image memorability highlights an apparent paradox around the nature of the signal that the brain is thought to rely upon to drive familiarity. Namely, several lines of evidence suggest that this signal is repetition suppression: the average reduction in the overall vigor of a population response when a stimulus is repeated compared to when it is novel^9–15^. In this scheme, the brain linearly decodes the population response on any given trial to guide behavior, where higher firing rates are decoded as novel and lower firing rates are decoded as repeated. In ITC, this account has been shown to align with behavioral signatures of familiarity, such as the rate of forgetting as time passes after an image is first seen^15^ (Fig. 1a).

The apparent paradox follows from the fact that image memorability is represented in ITC in the opposite way, where more memorable images produce more vigorous responses. When taking image memorability into account, the repetition suppression hypothesis fails to predict behavior (Fig 1b). This observation calls claims about the nature of the signal driving familiarity into question: if *reduced* population firing signals stronger memory, then how can more memorable images induce *greater* population firing (Fig. 1b)? While it’s tempting to propose that repetition suppression (the firing rate difference between novel and repeated images) drives familiarity, such explanations are ill-defined absent suggestions of how the brain would “remember” the ITC response to a novel image when viewing a repeated one such that a reference could be made to it. As such, it’s unclear how the brain solves this paradox in the transformation from visual representations into memory signals.

This paradox is not limited to memorability. Other visual properties of images, like contrast, also modulate ITC population magnitude in ways that cannot be reconciled with the claim that familiarity is signaled exclusively with ITC population response vigor^16^. One study attempted to reconcile a similar contradiction for image contrast by suggesting the existence of downstream computations that selectively extract memory information from ITC while discarding fluctuations attributed to image contrast. However, while that study proposed how ITC may be read out to resolve this paradox, it did not include recordings of downstream regions to confirm that these disambiguated signals are indeed present downstream of ITC in a manner consistent with the predictions of the suggested decoding scheme. There is reason to believe that the medial temporal lobe (MTL) may play a role: repetition suppression has been observed in primate HC when viewing images^17,18^, but it is not clear how it correlates with explicit reports of familiarity, or if it is influenced by memorability.

This study aims to fill this gap by recording from both ITC and HC to capture the contributions of downstream regions involved in the transformation from vision to memory and test the hypothesis that this paradox is resolved via computations that happen in the MTL. Here, we demonstrate that a resolution to the paradox is reflected in HC, where population response magnitude reflects an isolated visual familiarity signal that aligns with memorability behavior. We also show that the transformation from ITC to HC cannot be accounted for by trivial MTL computation (like thresholding ITC responses). Instead, our data are consistent with a description in which MTL computations linearly decode visual memory information from ITC in a manner that selectively disregards image-evoked population magnitude modulation.

## Results

### Single-exposure visual memory task

**Figure 2.**
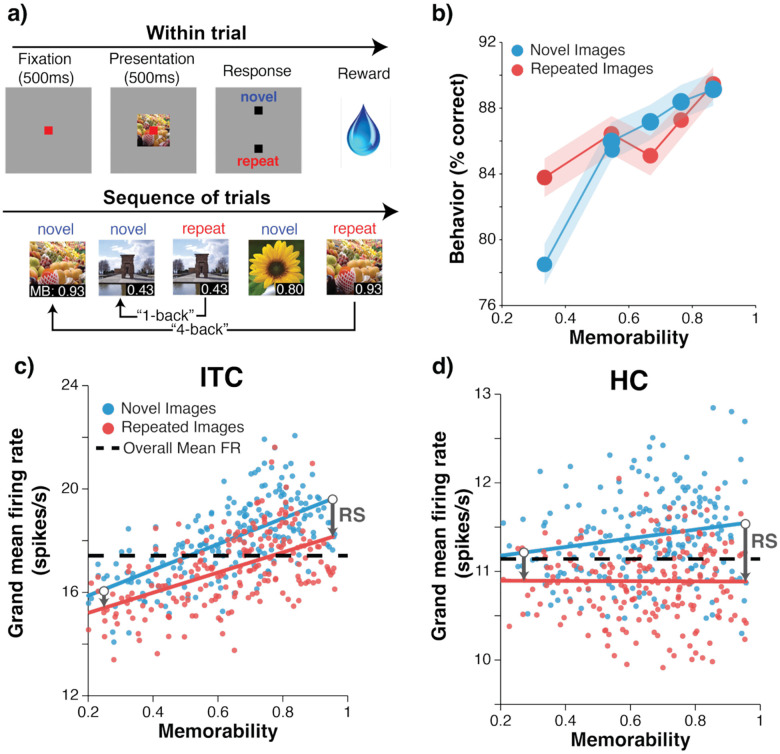
The familiarity-memorability paradox is resolved in HC. **a)** Two rhesus macaque monkeys performed a single-exposure visual recognition memory task in which they viewed one image per trial, each for 500ms, and responded with a saccade to a response target indicating whether they judged the image to be novel (never seen before) or repeated (seen exactly once before). Every image was shown exactly twice and the gap between novel and repeated presentations differed from immediate (“1-back”) to minutes (“64-back”). Images were drawn from a broad set of naturalistic objects and scenes and naturally varied in memorability, a scalar (range 0–1) that corresponds to the likelihood of a human correctly remembering that image in this task. Memorability scores were generated by using a convolutional neural network, MemNet, that was trained on human responses and that possesses accuracy approaching the ceiling imposed by inter-subject variability. **b)** The pooled results of the two macaque monkeys on this task for ‘novel’ (blue) and ‘repeat’ (red) trials (n=18,465 trials of each type). As predicted from the human-derived memorability scores, higher memorability images are more likely to be correctly recognized as repeated. Error shadow represents 95% confidence interval computed by bootstrapping (10,000 iterations). **c-d)** Neural data recorded in ITC (panel c) and HC (panel d) with spikes counted in the 300–500ms window following image presentation. Each dot represents the grand mean firing rate across all units to a novel (blue) or repeated (red) image of a given memorability. Average firing rate across all units and images is shown as a dashed line; this line would be the decision boundary in the most straightforward version of the repetition suppression hypothesis (any image above the line would be decoded as “novel”, and below the line would be decoded as repeated). In panel c (ITC), this decoding scheme would lead to poor predictions of observed memorability behavior (Fig. 1b), because higher memorability repeated images land above the line and would thus be classified as novel, however, these images are the most likely to be classified as repeated. In comparison, this decoding scheme applied to panel d (HC) would align much better with memorability behavior. Gray arrows indicate repetition suppression, which increases with memorability in both ITC and HC.

Two rhesus macaque monkeys performed a single-exposure visual recognition memory task (Fig. 2a). The monkeys sequentially viewed natural images varying in memorability and reported after each trial whether the image was novel (never seen before) or repeated (seen exactly once before) for a juice reward. Monkeys initiated a trial by fixating on a central fixation point. After fixating for 500ms, an image appeared for 500ms, subtending 4 degrees of the central visual field. After the image disappeared, the monkeys made a saccade to one of two response targets to indicate whether the image was novel or repeated. Correct responses were rewarded with juice. All images were shown exactly twice, and the time between the novel and the repeated presentation of an image varied from seconds to minutes (see Methods). After training, the monkeys performed this task proficiently (on repeated trials across all n-backs: Monkey 1 at 91% correct and Monkey 2 at 84% correct – Fig. S1). Images depicted a diverse range of categories and scenes and spanned a broad range of memorability scores (Fig. S2). Image memorability scores were extracted from a model designed to predict memorability for humans, MemNet^5^. This model was trained on human behavior and possesses accuracy near the ceiling imposed by inter-subject consistency. As previously reported^7^, these human-based memorability scores predict memory performance for monkeys, a finding replicated in these data (Fig. 2b).

### The memorability paradox is resolved in HC

As the monkeys performed this task, we recorded neural activity in ITC or HC with a 24-channel probe lowered acutely for each session. After screening for quality control (see Methods), the dataset included 7 sessions (290 units) and 16 sessions (312 units) for monkey 1 and 8 sessions (333 units) and 21 sessions (503 units) for monkey 2 in ITC and HC, respectively. We included units with activity that reflected task-responsiveness (see Methods), and images in which the monkey completed both the novel and repeated presentation trials. Because the responses of individual ITC and HC units are often tuned for specific visual features, estimating the overall population response requires many hundreds of units. Therefore, we concatenated units across sessions into a pseudopopulation and aligned images of similar memorability into “pseudoimages”. When creating these pseudopopulations, we aligned trials in a manner that preserved novelty, n-back, and approximate memorability scores (see Methods for details). Data were pooled between the two monkeys for each brain area.

As a replication of earlier work^7^, we began by examining the relationship between image memorability and the overall vigor of the ITC population response (Fig. 2b). We found a strong and highly significant correlation between memorability scores and grand mean firing rate (Fig. 2c; for repeated images: Pearson’s r(206) = 0.56, p=8.2e-29, novel images r(206) = 0.67, p=1.5e-18). Firing rates to repeated images were reduced by 7.6% on average relative to their novel presentations, reflecting repetition suppression.

In comparison, in HC the correlation between memorability and firing rate for repeated images was statistically indistinguishable from no relationship (Fig 2d; r(206) = −.01, p=.935) and was only modestly positive for novel images (r(206) = 0.20, p = .004). Average repetition suppression was a 4.64% reduction in HC. To statistically compare memorability modulation in ITC and HC, we fit linear regressions individually to both novel and repeated images in each brain area (solid lines on Fig. 2) and tested whether the slopes differed between ITC and HC via a permutation test of the null hypothesis that the slope of the MB vs GMFR relationship was the same in ITC and HC (see Methods). We found that memorability modulation was significantly attenuated in HC as compared to ITC for both repeated (ITC β=4.983 and HC β=0.48, (H0: Δ β = 0, p = .0047) and novel (ITC β=3.908, HC β=-0.012, (H0: Δ β = 0, p = .0034) images.

**Figure 3.**
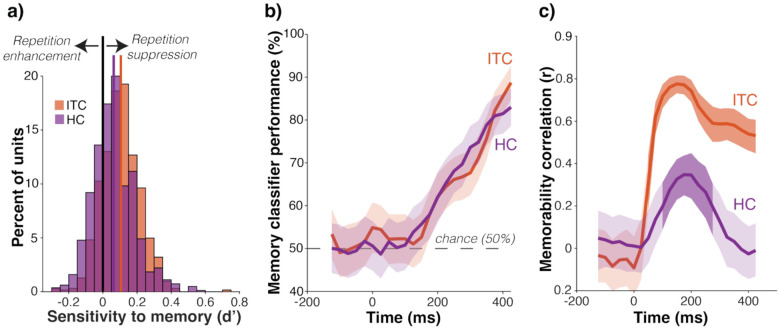
The data are consistent with a feedforward transformation between ITC and HC **a)** The distribution of sensitivity to memory (d’) across the units in the ITC and HC populations (602 units in ITC, 836 in HC) in the 300–500ms spike count window. On the x-axis, a positive value for repetition suppression corresponds to units that fire less to a repeated presentation than to a novel one. The solid black vertical line separates units that are enhanced by repetition (left) from ones that are suppressed (right). **b)** Time course of memory signal in ITC and HC measured by the performance of a weighted linear decoder as a function of time (150ms spike count window, 20ms sliding intervals). Shaded regions represent one standard deviation across cross validations. **c)** Time course of the correlation between memorability and grand mean firing rate in ITC and HC (150ms spike count window, 20ms sliding intervals). Shaded regions reflect 95% confidence intervals, computed by bootstrapping with 10,000 resamples. Darker shadows indicate times in which the correlation is significantly different from zero (p<.05).

The significant attenuation of memorability modulation in HC relative to ITC held for both monkeys individually (Fig. S3) and when considering a range of spike-count windows following stimulus onset (Fig. 3C). In both ITC and HC, repetition suppression increased as a function of memorability (emphasized with gray arrows in Fig. 2c–d and quantified in Fig. S4).

To summarize, these results replicate prior observations that both repetition and memorability modulate population response vigor in ITC. As such, memory cannot be reflected in ITC in a manner that can be decoded from population response vigor alone (Fig. 1b, Fig. 2c). These results establish for the first time that a solution to the familiarity-memorability paradox is reflected in the population response vigor in HC, where population vigor reflects a largely isolated representation of memory in which repetition suppression is larger for more memorable images, but population vigor is otherwise largely unaffected by memorability.

### The data are consistent with a feedforward transformation between ITC and HC

We hypothesized that these results reflect computations downstream of ITC that reformat information somewhere in the medial temporal lobe as it propagates from ITC to HC. To evaluate the plausibility of this scenario, we examined the amount, timing, and format of memory information in ITC and HC to determine if it was consistent with this hypothesis.

Carrying memory forward as repetition suppression would be the simplest to implement for a feedforward computation, so we first explored the nature of memory information in both ITC and HC. Specifically, we determined whether memory was reflected in individual units as repetition suppression, repetition enhancement, or some combination. If memory is reflected in individual units in HC as repetition suppression, this would be consistent with a straightforward feedforward account. We tested this by computing sensitivity to memory (d’) for individual units and comparing the distributions in ITC and HC. Histograms of d’ in both brain areas were roughly normal but shifted towards positive values (repetition suppression) in both HC and ITC, indicating that both populations were dominated by repetition suppression along with some noise (Fig. 3a). We verified that the small fraction of repetition enhanced units in both brain regions were in fact noise with a ranked population decoding analysis (Fig. S5). These results suggest that, like ITC, the nature of the memory signal in HC is repetition suppression.

A feedforward account predicts that memory information will arrive in ITC before (or nearly the same time) as HC. To test this prediction, we compared the temporal dynamics of memory information in ITC and HC. Specifically, we trained and tested a cross-validated FLD memory classifier (see Methods) along a sliding window aligned to stimulus presentation. Consistent with the feedforward predictions, memory information rises at approximately the same time in HC as ITC (Fig. 3b). As such, the temporal dynamics are not at odds with the proposal that memory information is fed forward from ITC to HC.

In sum, like ITC, the HC population represents memory information in the form of repetition suppression, and this suppression is reflected in ITC and HC at approximately the same time following stimulus onset. Taken together, these results are consistent with a feed-forward description in which memory and memorability both modulate ITC population response vigor, and medial temporal lobe computations eliminate (nuisance) memorability modulation in a manner that preserves memory, reflected unambiguously as population response vigor in HC.

### The transformation between ITC and HC is non-trivial

We next asked if, instead of a downstream computation, intertwined memory and memorability modulation could be resolved within ITC itself over time by the end of the 500ms viewing period, with a ‘late readout’ being propagated forward to HC. We found that within HC, the correlation of memorability and mean firing rate drops to levels indistinguishable from no correlation by approximately 300ms following stimulus onset. In contrast, it remains highly correlated within ITC throughout the entire viewing period (Fig. 3c). Consequently, a late readout of ITC alone cannot explain the transformation from ITC to HC.

Following on evidence that visual representations in HC^19^ are thought to be sparse and those in ITC^20^ are less so, we wondered if the transformation from ITC to HC could be described by simple thresholding. Specifically, we tested whether HC units decoding ITC units with a threshold that chopped off the flanks of ITC tuning curves could account for the transformation observed in the neural data.

**Figure 4:**
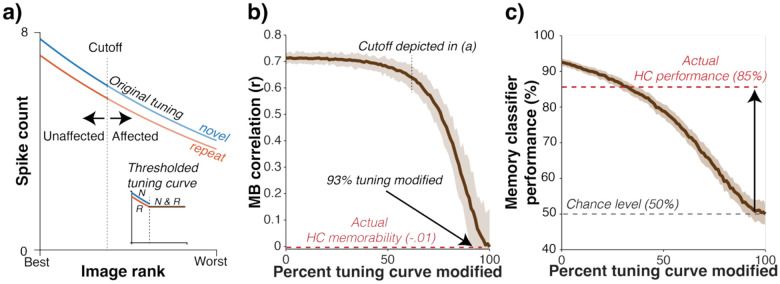
Thresholding ITC cannot explain the transformation to HC. **a)** To evaluate the plausibility of the thresholding proposal, we performed a simulation based on tuning curves fit to each unit. Shown is an example unit. The red and blue lines correspond to the firing of this unit to novel and repeated images, arranged from best (highest evoked firing) to worst (lowest evoked firing) images on the x-axis. The vertical dashed line denotes the threshold at which the tuning curve is modified, and the inset shows the tuning curve after the modification. To assess the impact of thresholding, the same threshold (e.g., 60% as shown in panel a is applied to all ITC units, and the modified tuning curves are used to generate a simulated population; this is then repeated for a range of thresholds. **b)** The correlation between memorability and GMFR for the simulated populations is plotted as a function of the percent of the tuning curves that were modified on all units. To match the observed memorability correlation in HC, 93% of the tuning curve would have to be thresholded away. Error shadow depicts the 95% confidence interval. **c)** Memory information remaining in each simulated population after thresholding, assessed by a weighted linear decoder. The threshold required to match memorability (panel b) destroys nearly all memory information, implying that this proposal is implausible.

To evaluate this proposal, we performed a simulation by fitting a model to each ITC unit that describes its visual tuning and memory sensitivity (Fig. 4a, see Methods). We confirmed that the model ITC population recapitulated salient aspects of our data, including the correlation between population response vigor and memorability (Fig. S6). Next, we applied the thresholding operation described in Fig. 4a, where N% of the peak of each unit’s tuning function was set to a threshold, explored for the full range of N (0–100%). The resulting model populations were analyzed for both their memorability correlations (Fig. 4b) and memory classifier performance (Fig. 4c).

We found that matching the observed HC memorability correlation required thresholding a remarkable 93% of ITC tuning functions (Fig. 4b). The consequence of this amount of thresholding was the elimination of nearly all memory information, at odds with the actual HC population whose memory performance is matched to ITC (Fig. 4c). As such, simple thresholding (leading to sparsification) cannot account for the transformation from ITC to HC.

Next, we considered a different proposal in which the transformation from ITC to HC happens via a decoding scheme that seeks to eliminate memorability while preserving memory information. A previous study^16^ proposed this type of mechanism for a similar challenge where the confounding variable was contrast, which also modulates ITC firing rate vigor. However, unlike memorability, memory behavior in that study was largely unaffected by the confounding variable (contrast), and this decoding scheme thus operated by maintaining the same memory performance regardless of contrast. As such, it is unclear whether the proposed solution could also work for memorability, where behavior varies systematically as a function of the confounding variable. In the case of memorability, the decoding scheme applied to ITC would need to eliminate memorability modulations while maintaining the relationship between repetition suppression and memorability to predict behavior.

The fact that both memorability and memory modulate the overall firing rate of ITC suggests that they probably both modulate the same ITC units (as opposed to nonoverlapping subpopulations that are independently modulated by one or the other). The decoding scheme we evaluate here proposes that as long as representations of memorability and memory are not perfectly overlapping (i.e., influencing the firing rate of every unit equally), each can be decoded separately from the other. The question then becomes: can this decoding scheme account for the transformation from ITC to HC, or does it happen at the cost of such memory information loss that it (like the threshold mechanism) is implausible?

**Figure 5.**
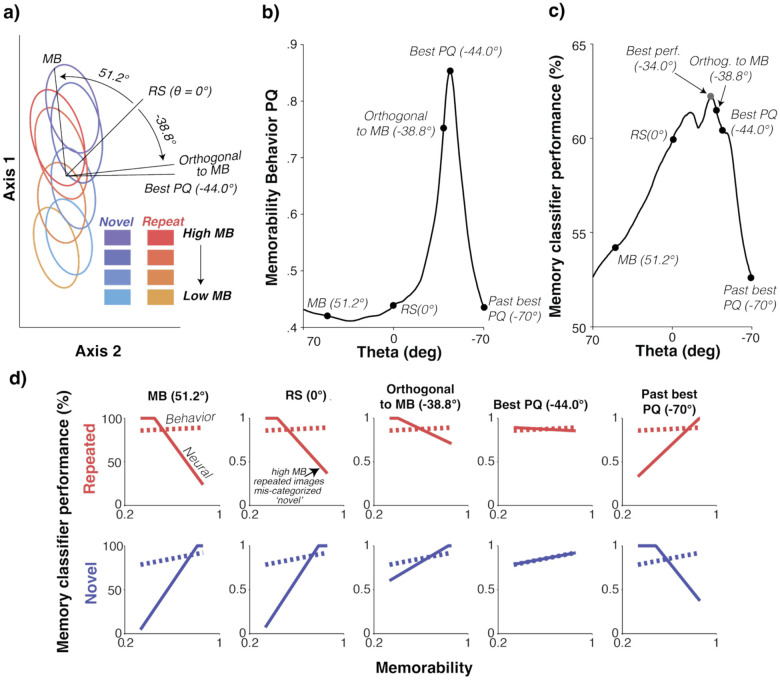
A memorability-correcting decoder applied to ITC predicts behavior. **a)** Projections of ITC neural data onto a plane defined by repetition suppression (RS, where every unit is weighted equally) and a memorability decoder (MB, optimized to decode high versus low memorability images), rotated 45 degrees for clarity. Ellipses indicate the one-standard-deviation contour for 2D histograms of the projections of ITC population responses onto this plane. The eight ellipses correspond to novel (blues) and repeated (reds) images grouped by memorability scores into quadrants (hue). The classifier axis that best predicts behavior sits ~5 deg away from orthogonal to MB decoder (i.e. the one with no sensitivity to memorability). **b)** The quality of prediction (normalized difference in angle between the slope of the predicted to actual behavior, average of novel and repeated predictions) for different linear decoders defined by their rotation within the plane relative to the vector that weights all units equally (1,1,1,….). **c)** FLD Memory classifier performance at different angles of rotation within the MB/RS classifier plane (average of novel and repeated performance). **d)** Comparison of behavior (dashed) with neural predictions (solid) for five decoder rotations. For visualization, the neural predictions are rescaled to the same range as behavior with a multiplicative factor, loosely consistent with an adjustment in population size (see Methods). All analyses were performed in the 100–500ms spike count window following image presentation.

To assess the plausibility of this proposal, we defined two classifiers: a repetition suppression-based (RS) classifier weighting each unit equally (the 1,1,1, … vector) that separates novel from repeated images, and a memorability classifier (MB) that separates images with high versus low memorability (see Methods). These decoding axes were 51 degrees apart in the plane defined by them (Fig. 5a), confirming that they were overlapping (relative to the benchmark of orthogonality, 90 degrees), but only partially so.

To decode memory independent of memorability, we considered vectors on this plane (Fig. 5a). We quantified the ability of the full suite of linear decoders that populated this plane to account for behavior with a prediction quality (PQ) metric that quantified the alignment of these predictions to behavior, averaged for novel and repeated images. As shown in Fig 5d, sweeping through this plane starting with MB and RS produced ITC decoded behavioral predictions that began misaligned with behavior, became aligned, and were then misaligned once again (but in the opposite direction). We identified the decoder in this plane that most accurately predicts behavioral performance as a function of memorability and labeled it the point of “best PQ” (Fig 5b,d).

The best PQ fell nearly orthogonal to the MB classifier, at 95.2 deg away (Fig 5b). Intuitively, this makes sense because the goal is to extract memory information largely independent of memorability. The fact that the best PQ vector falls a bit past 90 degrees aligns with the small amount of memorability modulation reflected behaviorally.

Above, we ruled out thresholding as a mechanism to describe the transformation between ITC and HC, with the rationale that the amount of thresholding required would destroy nearly all memory information (Fig. 4). In comparison, with this mechanism, memory information is largely retained (Fig. 5c), and performance is higher with the Best PQ classifier than it is with the RS (1,1,1,…) classifier. The fact that it is not the highest of all possible classifiers on this plane reflects a small trade-off between the preservation of the memory information reflected in ITC and the elimination of memorability modulation reflected as population response vigor.

In sum, these results suggest that while both memory and memorability information are overlapping in ITC, that overlap is partial. As such, a linear decoding scheme can be designed to selectively decode memory while attenuating memorability. We found that a decoder of this type provided a good account of the transformation from ITC neural responses to memorability behavior. When combined with the finding that a resolution to the familiarity-memorability paradox is reflected in HC (Fig 2), these results suggest that the MTL may apply this type of computation to ITC to selectively extract memory signals to drive behavior.

## Discussion:

Here, we leverage the systematic variability with which humans and monkeys find the same images memorable to understand how seeing is transformed into remembering. Namely, the fact that more memorable images produce more vigorous responses in ITC challenges the hypothesis that memory can be decoded from firing rate in ITC, following on the observation that repetition suppresses firing to repeated images. Memorability (along with other visual features of images such as contrast^16^ and size^21^) modulates the vigor of the ITC neural response, and these stimulus-induced modulations of population vigor catastrophically interfere with hypothetical magnitude-based memory signals. We find that for the case of memorability, this paradoxical representation is solved downstream of ITC, in hippocampus.

The finding that the hippocampus reflects an isolated visual memory representation in which memory signals are stronger for more memorable images alone does not answer how this computation is performed. One misleading suggestion is that repetition suppression itself (the firing rate difference between novel and repeated images) can be somehow decoded from ITC directly. However, such explanations are ill-defined absent suggestions of how the brain would “remember” the ITC response to a novel image when viewing a repeated one such that a reference could be made to it, leaving open the question of how the brain solves this paradox to transform visual representations into memory signals. Here, we demonstrate that this familiarity-memorability paradox cannot be resolved trivially, such as by a ‘late readout’ of ITC (Fig. 3c) or via simple thresholding (Fig. 4). However, it can be explained by a linear decoding scheme applied to ITC that selectively extracts memory information while disregarding image-evoked population magnitude modulation. Further, we demonstrate that a resolution to the paradox is reflected in HC, suggesting that the MTL may perform this decoding computation.

**Figure 6:**
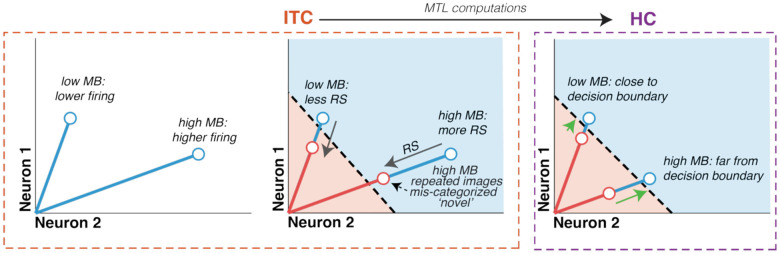
Schematic of the proposed two-stage process that converts visual experiences into memories. Left: In the first stage (in ITC, orange dashed box), images evoke patterns of spikes coding for their identity, corresponding to population vector angle, and memorability determines population vigor, corresponding to population vector length (ITC left, blue). Middle: When an image is repeated (red), it triggers a reduction in firing, repetition suppression (RS, gray arrows). This creates larger magnitude RS for more memorable images because they evoke stronger novel firing. However, memory cannot be decoded from population response vigor in ITC, because highly memorable images fall on the wrong side of the decision boundary (dashed black line, blue shaded region would be decoded as ‘novel’ and red shaded area as ‘repeated’). Next, this representation undergoes a transformation in the medial temporal lobe to attenuate memorability modulation while retaining RS proportional to memorability. As a result, in HC (right, purple dashed box), the repeated presentations of higher memorability images are further (green arrow) from a decision boundary based on the overall mean firing rate. Consequently, a decoder can classify a neural representation as deriving from a novel or repeated image and explain memorability behavior by simply decoding overall firing rate.

Our results suggest a simple account of how the transformation from seeing to remembering happens. First, more memorable images produce more vigorous responses in ITC (Fig. 6, left). This, in turn, causes more repetition suppression (Fig. 6, middle). For instance, if repetition suppression were a proportional reduction in firing rate, more vigorous responses would produce larger magnitude reductions (as an illustration, 10% of 100 is 10; 10% of 10 is 1). In this study, we found repetition suppression to be modestly supra-proportional, meaning that the more vigorous firing associated with more memorable images produced slightly more repetition suppression than predicted by a constant, proportional reduction (Fig. S4).

Next, because memorability modulates ITC firing, memory cannot be unambiguously decoded from population response vigor, leading to the familiarity-memorability paradox (Fig 6 middle, dashed line; also described in Fig. 1). Computations in medial temporal lobe are thus required to disambiguate ITC modulations of population vigor from sources other than memory, leaving an isolated memory representation reflected as population response vigor in HC (Fig. 6, right). We present two key pieces of evidence consistent with this understanding: (1) Downstream of ITC, in HC, memorability modulation is largely attenuated but memory modulation remains, leaving familiarity in the form of RS in HC (Fig 2); and (2) a decoding scheme of this type applied to ITC maps to memorability behavior (Fig 5).

This proposal sheds light on how the brain stores so many memories at high fidelity^22,23^. Here, image memories are stored within ITC via a coding scheme that complements image identity representations (reflected as patterns of activity across a population) by modulating population response vigor. The medial temporal lobe then contributes by disambiguating memory information from other sources that modulate population vigor.

We found that a memorability-attenuating linear decoding scheme applied to the ITC population could decode memory in a manner that captures memorability behavior. For this scheme to work, there must be meaningful heterogeneity in the sensitivities of individual ITC units to memory and memorability (or their representations would completely overlap). That the decoding scheme in Fig. 5 faithfully maps ITC responses to behavior reveals that this heterogeneity exists in ITC, and it suggests that the medial temporal lobe could use these differences to disambiguate memory from memorability modulation and discard memorability nuisance modulation. A similar proposal was previously suggested for image-contrast^16^, but in that case, behavioral performance was matched for images that produced higher versus lower firing rates (modulated by contrast). Here, we show that a memorability-attenuating decoder applied to ITC can recapitulate higher behavioral performance for more memorable images. We also add the crucial observation that a transformation like this does happen downstream of ITC, such that the vigor of the HC population response is modulated by memory (not memorability), but larger memory signals for more memorable images remain.

Future work will be required to determine how the medial temporal lobe might learn to decode ITC in this way. The memorability classifier used in Fig. 5 was trained on outside knowledge of which images had high or low memorability, but humans do not have naive conscious access to how memorable images are^3^. However, memorability follows defined statistics^4,24^, which the brain could learn. Underscoring this point, neural networks trained to predict memorability are able to do so for unseen images^1,25^, and the brain could learn these statistics as well.

While the simulations shown in Fig. 4 show that a high-thresholded-readout (something that might generate the sparsity that supports decorrelation and pattern separation in HC) cannot by itself lead to the attenuated memorability representations, our results do not rule out other schemes. For instance, several lines of evidence suggest that the dentate gyrus of HC performs “pattern separation” computations to sparsify sensory representations arriving from medial temporal lobe structures such as entorhinal cortex^26,27^. Likewise, hippocampal place cell-like representations can arise via an autoencoder-based scheme designed to compress memories^28^. The attenuation of memorability observed in HC relative to ITC could conceivably be linked to these other computations.

Individuals with damage linked to HC, including mild cognitive impairment, have long been thought to perform normally on recognition memory tasks (as long as the images are highly distinct^29^). However, recent investigations have demonstrated memorability deficits in individuals with mild cognitive impairment^30^. Of note is that in that study, it was unclear what (or whether) there was a specific neuroanatomical deficit in these patient populations. Future work investigating memorability deficits in individuals with circumscribed damage (such as the hippocampus) could be informative. One prediction from our result is that damage to the mechanism that attenuates memorability should produce a reversed behavioral pattern, whereby images that are typically the most memorable should be the least memorable (Fig. 1).

Understanding how the healthy brain transforms experiences into memories is foundational for understanding memory-related disorders. Here we resolve a paradox related to how that transformation happens, focusing on the nature of the neural code that the brain uses to drive reports and how its underlying neural signals come to be, and we present evidence for an emerging hypothesis of how visual memories are stored and signaled.

## Methods:

All data were collected from two adult rhesus macaque monkeys (*Macaca mulatta*). Monkey 1 was male and monkey 2 was female. All procedures were performed in accordance with the guidelines of the University of Pennsylvania Institutional Animal Care and Use Committee.

### Anatomy and recording:

The activity of neurons in ITC and HC were recorded via a single recording chamber (Crist Instruments, Hagerstown, MD) in each monkey. In monkey 1, we recorded from left ITC and HC. In monkey 2, we recorded from right ITC and HC. Chamber placement was guided based on anatomical magnetic resonance imaging. ITC and HC were recorded in separate sessions. Neural signals were recorded with 24-channel U-probes (Plexon Inc., Dallas, TX) with recording sites spaced at 100 μm intervals. Wideband signals were amplified and digitized at 30 kHz using a Grapevine Data Acquisition System (Ripple, Inc., Salt Lake City, UT). Spikes were hand-sorted offline (Plexon Offline Sorter).

The region of ITC recorded was located on the ventral surface of the brain, located 17 mm lateral to the sagittal plane defined by the central sulcus and 13–16mm anterior to the coronal plane defined by the ear canals.

The region of HC recorded was 11 mm lateral to the sagittal plane defined by the central sulcus and 11–16 mm anterior to the coronal plane defined by the ear canals, and only includes areas roughly in the dorsal half of HC (this targeting was guided by ref^17^). This means that our sample predominately includes hippocampal subfields CA3, DG and CA4 (approximately in that order of relative prevalence) but because of the organization of the primate hippocampus and the inherent ~+/− 1 mm level of precision in our recording preparation, we do not attempt to distinguish between the subfields. However, because subfield CA1 is located ventrally in primate hippocampus and we targeted our recordings to the dorsal half of HC, we are reasonably confident it is not represented in these populations.

### Inclusion Criteria:

A multi-channel recording session was included in the analysis if (1) there were no technical problems with the recording hardware, (2), it fell within the anatomical boundaries described above, (3) the monkey completed at least 200 novel-repeat image-pairs during the session, (4) the session was stable, defined as the overall baseline firing rates across all channels did not change by more than 2-fold across the session, and there was no significant difference between baseline firing rates preceding novel and repeated images (t-test, p<.01, in the 500ms prior to image presentation) and (5) the session was composed of over 50% visually responsive neurons (in ITC) or contained at least a single visually responsive neuron (HC), determined by an inspection of the raster plots. We chose this relatively strict criterion for ITC to ensure high-quality data based on our previous experience recording this highly visual brain area, and a much looser criterion for HC because we did not wish to make any *a priori* assumptions about its visual organization. Approximate memory information was matched between populations by truncating a handful of sessions (criterion 6). Within a session, units were included in the population if they evoked a response in the post-stimulus window [50–350ms] versus the pre-stimulus [-300,0] window (two-sided t-test, p<0.10) across all trials in the session. In monkey 1, this filter passed 96% and 52% of units for ITC and HC respectively, and for monkey 2 this filter passed 93% and 57% of units for ITC and HC respectively.

Monkey 1’s ITC population was collected over the course of approximately five weeks. We collected 20 sessions and 13 were removed (1, 1, 1, 6, and 4 from criteria 1, 3, 4, 5 and 6 respectively).

Monkey 1’s HC population was collected intermittently over the course of approximately seven months. We collected 36 sessions and 20 were removed (3, 8, 3, and 6 from criteria 1, 3, 4 and 5 respectively)

Monkey 2’s ITC population was collected intermittently over the course of approximately four months. We collected 9 sessions and 1 was removed (from criteria 1)

Monkey 2’s HC population was collected over the course of approximately two months. We collected 30 sessions and 9 were removed (2, 3, 2, 1, and 1 from criteria 1, 2, 3, 4, and 5 respectively)

### Behavioral task:

A custom MWorks script (The MWorks Project, Cambridge, MA) was used to display images on an LCD monitor and collect behavioral responses. The experiment was performed in a darkened testing chamber with the monkeys’ head fixed while their gaze was tracked with an Eyelink 1000 (SR Research, Ottowa Canada). Trials were initiated by the monkey fixating on a red square fixation point (0.35 degrees) in the center of a gray screen within an invisible square window (4 degrees monkey 1, 3.5 degrees monkey 2), followed by a 500ms delay before a 4 degree image appeared on the center of the screen. The monkeys had to maintain fixation on the fixation point for 500ms, after which the image disappeared from the screen and the fixation point changed to a green go cue, and two square white .5 degree response targets appeared 8 degrees above and below the stimulus. The monkey indicated a response by making a saccade to one of the two targets. If the monkey was correct, they received a liquid reward. For Monkey 1, the target associated with “novel” images was the top target; this was reversed for Monkey 2. If the monkey broke fixation, the image immediately disappeared and a new trial was offered after a short delay. In cases where the stimulus was novel but the monkey broke fixation (after an image had appeared on the screen), the subsequent presentation of that image was still rewarded as a “repeat,” although incomplete image pairs were excluded from further analysis.

A custom MATLAB (The MathWorks, Inc. Natick MA) script was used to pseudo-randomly generate a sequence of images, where each image appeared exactly twice (the same algorithm as ref^15^). The script aimed to match a target distribution of n-back trials. To fill the sequence, some occasional off-target n-backs were used but were not included in analysis. Due to behavioral differences between the two monkeys, the exact n-back distributions varied between Monkey 1 and Monkey 2.

Monkey 1 was presented with n-back trials of 1, 2, 4, 8, 32, 48, 64, and 192. The distribution was uniform for n-backs between 2 and 64, while 1 and 192 were half as frequent (e.g., in a sequence, there could be 15 1-back trials, 30 2-back trials, 30 4-back trials, and so on).

Monkey 2’s sequence included n-backs of 1, 2, 4, 8, 16, 32, 48, and 64, with a uniform distribution between 2 and 48 and half-frequencies for 1 and 64. N-backs differed between monkeys in order to approximately match performance between animals.

The images used were drawn from the same set used in Jaegle et al. (2019)^7^, and images used during training a monkey were never reused during recording.

### Behavior:

In Fig. 2, all trials eligible for inclusion in the dataset were used to plot the monkey’s behavior. In Fig. 4, only trials selected for the pseudopopulation were included for determining the behavioral benchmark that neural predictions were compared to.

### Pseudopopulation:

In the pseudopopulation, a response to a given unit always contained the novel/repeated pairing of the same real images, and pairs were aligned so that a pseudoimage always included a similar n-back separation and images with similar memorability scores. We also used these alignments to pool the data between monkeys. This process of pooling and aligning limits the number of images included in the pseudopopulation to the number of eligible images in the shortest recording session, and therefore some trials from other sessions were discarded. This was done pseudo-randomly and not biased to any experimental conditions.

### Permutation test for difference in slopes (Fig. 2):

To perform this test, a null hypothesis distribution was generated by randomly assigning an “IT” or “HC” label to each data point, without replacement, then fitting lines to these shuffled “populations” and calculating the difference in slopes between the two “brain areas”. This procedure was repeated 10,000 times to generate a distribution of the null hypothesis that there was no difference between the brain areas. P-values were calculated by computing the proportion of null-distribution slopes that differed from the actual slope.

### RS and FLD classifiers:

These classifiers were cross validated and took the form of linear discriminators in which the class of a population response vector to a pseudoimage was determined by the sign of:

f(x)=w⋅x-b

Where **x** is the population response vector, **w** is an n-dimensional weights vector in the n-dimensional ITC or HC neural space (n being the number of units) and b is a decision boundary given by:

$$b=\frac{1}{2}*w\cdot \left(\mu_{1}+u_{2}\right)$$

Where *μ*_1_ and *u*_2_ are the means to the two classes (novel and repeated).

For the FLD decoder we used the same implementation as our previous work^15,16^:

$$W_{FLD}=\Sigma^{-1}\left(\mu_{n}-\mu_{r}\right)$$

Where $\Sigma^{-1}$ is the inverse of the covariance matrix averaged across both conditions. Because the dimensionality of the neural data is very high relative to our sample size, we cannot make good estimates of the off-diagonal terms in the covariance matrix so they are set to zero. This results in the classifier effectively weighting each unit by its d’.

Because memorability confuses classifiers of this type (as described in Fig. 1), for the implementation of the FLD classifier in Fig. 3 and S5, the pseudopopulation alignment by memorability was broken by randomly shuffling the images that compose pseudoimages (within each unit). This means that every pseudoimage was composed of random images, ameliorating the influence of memorability.

In Fig 5c, this alignment of images was retained, leading to the relatively lower performance.

### Ranked FLD (Fig. S5):

The same implementation of the FLD classifier described above was used. Weights for all units were calculated within each cross-validation iteration, then units were ranked by those weights, subsets of units were removed, and the weights (and threshold) were re-calculated before testing the classifier.

### Population fits/synthetic population generation and modification (Fig. 4):

We fit units following an adaptation of the method described in ref^16^. In brief, this involved fitting a three-parameter exponential model for each unit that maximized the likelihood of observing the spike count data from 100–500ms:

$$y(x;M)=A_{M}e^{-\alpha x}$$

Where *x* is the stimulus rank along the tuning curve, M is the memory condition (novel or repeated), A is amplitude (*A*_*N*_ and *A*_*R*_, for novel and repeated images, respectively, fit separately), and *α* controls the sharpness of the curve (to capture image selectivity).

To generate synthetic populations based on our observed data, we ranked the observed spike counts for each image seen (averaging across novel and repeated presentations) to get an *x* value (image rank) for each image for each unit. These values were then used to solve for a predicted spike count for a given image and unit using the fit constants for *A*_*N*,_, *A*_*R*_ and *α*. We then used a Poisson process to generate a synthetic spike count. Memorability in these simulations derive from the memorability scores of the originally shown images

To generate the ‘modified populations in Fig. 4b–c, the predicted firing rate for both novel and repeated was changed to that of the repeated firing for any image ranked lower on the tuning curve than the ‘cutoff point’ rank.

### Memorability-corrected linear decoder:

To train and test a decoder that might account for the transformation between ITC and HC (Fig. 5), we identified RS and MB classifiers (for the same training data).

The RS decoder evenly weighted each unit so that:

$$W_{RS}=[1,1,1\ldots ]$$

The MB decoder was a “prototype classifier” such that:

$$W_{MB}=\mu_{Hmb}-\mu_{Lmb}$$

Where $\mu_{Hmb}$ is the mean response to images in the top 50% of memorability scores and $\mu_{Lmb}$ is the mean responses to images in the bottom 50% of memorability scores.

The classifiers rotated within the plane formed took the form of:

$$W(\theta )=(\text{cos}\;\theta -\text{cot}\gamma \;\text{sin}\;\theta )\overset{ˆ}{1}+(\text{csc}\;\gamma \;\text{sin}\;\theta )\;{\hat{w}}_{MB}$$

Where $\overset{ˆ}{1}$ is the unit vector representing the RS axis and ${\hat{w}}_{MB}$ is the unit vector representing the MB classifier and *γ* is the angle between the MB and RS axes.

### Projections onto plane defined by $\hat{1}$ and ${\hat{w}}_{\text{MB}}$ and error ellipses (Fig. 5a):

The data used to visualize the contour ellipses were the same held-out test set used to train the MB and RS decoders, split into four divisions by memorability quartile boundaries. The ellipses were computed by projecting the data onto the non-orthogonal $\overset{ˆ}{1}$ and ${\hat{w}}_{MB}$ axes. This coordinate system was then rotated by 45 deg (within the plane) to visualize the RS classifier at this angle. For each condition, the covariance matrix of transformed data was computed, and eigenvectors of this matrix provided the major and minor axes of the associated ellipse. The ellipses themselves represent one standard deviation contours of the 2-dimensional histograms projected onto this plane.

### Prediction quality (Fig. 5b):

To determine the quality of the prediction between actual behavior and that predicted by ITC decoded performance, we divided the testing set for each round of cross-validation into four memorability bins with the quartile boundaries defined by the quartiles of the overall memorability distribution (these were also used to plot the contours, see above). They were further divided by novel and repeated images, and novel and repeated prediction qualities were calculated separately. These test sets were used to create ITC decoded predictions of behavior. To quantify prediction quality (PQ), we fit a linear regression to both the behavioral and neural predicted data and compared the slopes of the regressions such that:

$$PQ=1-\frac{\Delta \theta }{90^{\circ }}$$

Where *θ* is the angle between the behavioral and neural-predicted slopes. Because slopes separated by 180 degrees are equivalent, any angular difference greater than 90° was reflected across the 90° deg axis by replacing any difference greater than 90° with 180 − Δ*θ*. This metric yields a value of 1 when the slopes are perfectly aligned and 0 when they are perpendicular. The final PQ plotted in Fig 5b is an average of the novel and repeated PQs.

### Behavioral predictions based on classifiers (Fig. 5d):

Any comparison between population decoded predictions and actual behavior requires implicitly or explicitly specifying the number of neurons included in the decoding analysis. Following on ref^16^, we confirmed that performance using all recorded units in our dataset fell below saturation, and then simulated increases in population size by fitting a single rescaling parameter by solving for a single multiplicative scaling factor (i.e. one that does not change the slope of the line) that minimizes the squared error between the mean neural prediction and mean behavior. Any rescaled predictions over 100% were clamped to 100.

## Supplementary Material

1
