# Supplementary material for: Resolving a paradox about how vision is transformed into familiarity
Source: bioRxiv. 2025 Jun 14:2025.06.13.659490. Preprint. [Version 1] doi: 10.1101/2025.06.13.659490 (PMC12259176; doi:10.1101/2025.06.13.659490)
Supplement: 1 [file NIHPP2025.06.13.659490V1-supplement-1.pdf]

bioRxiv preprint doi: https://doi.org/10.1101/2025.06.13.659490; this version posted June 15, 2025. The copyright holder for this preprint (which was not certified by peer review) is the author/funder, who has granted bioRxiv a license to display the preprint in perpetuity. It is made available under aCC-BY 4.0 International license.

Supplementary Figures:

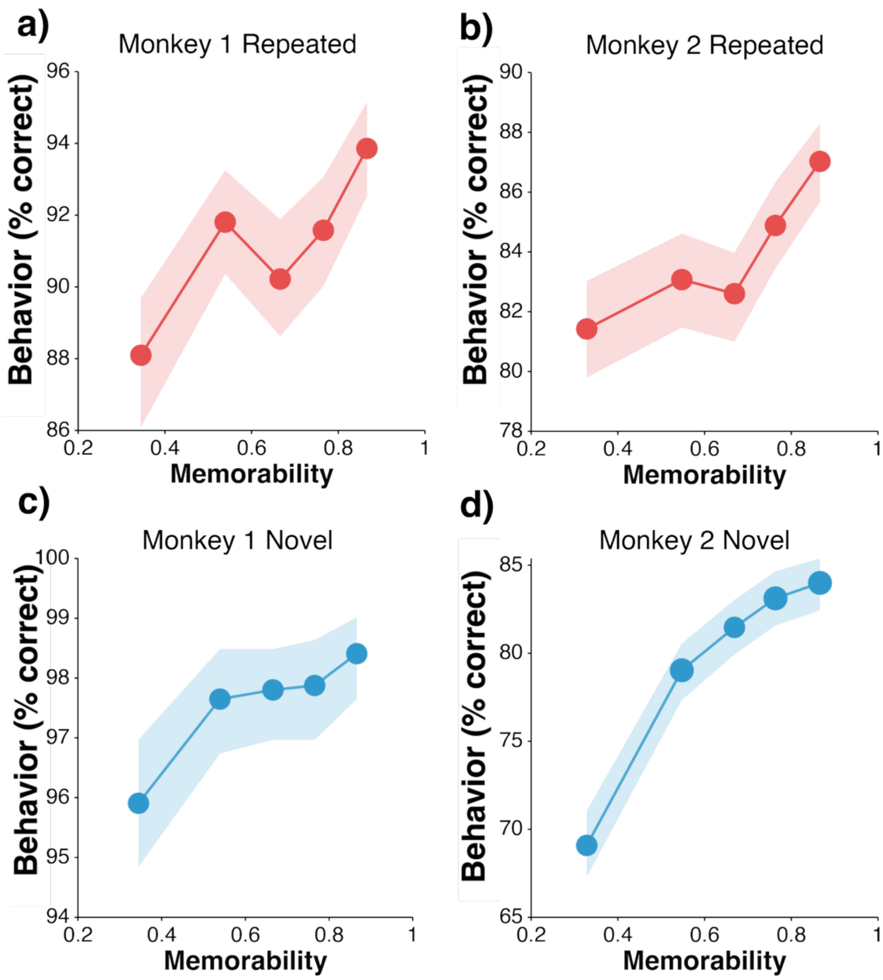

Figure S1*: Individual monkey behavior. (*a-b) Percent correct on repeated trials for monkey 1 (a; n=6,593 trials, 91.1% mean) and monkey 2 (b; n=11,872 trials, 83.8% mean). (c-d) Percent correct on novel trials for monkey 1 (c; n=6,593 trials, 97.5% mean) and monkey 2 (d; n=11,872 trials, 79.3% mean). Shaded regions represent 95% confidence intervals calculated by bootstrapping with 10,000 resamples.

bioRxiv preprint doi: https://doi.org/10.1101/2025.06.13.659490; this version posted June 15, 2025. The copyright holder for this preprint (which was not certified by peer review) is the author/funder, who has granted bioRxiv a license to display the preprint in perpetuity. It is made available under aCC-BY 4.0 International license.

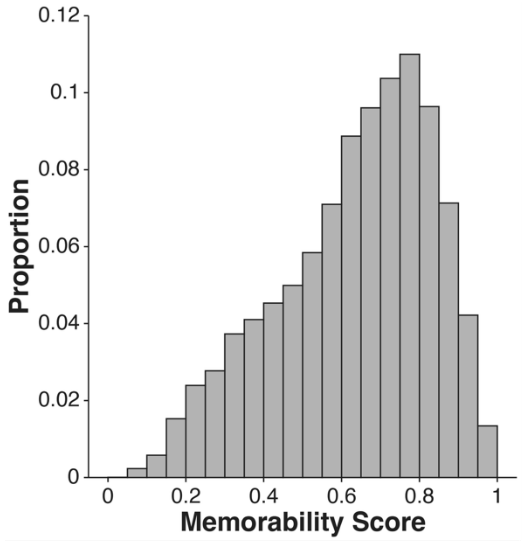

Figure S2: *Distribution of memorability scores across all images.* Shown for all images in which one or the other monkey completed both the novel and repeated trial (n=17,537 images). Images were scraped from the internet and covered a wide variety of objects and scenes. They were presented in full color. Memorability scores were acquired using the convolutional network MemNet[5], which is trained to predict human visual recognition memory on a similar visual recognition memory task.

bioRxiv preprint doi: https://doi.org/10.1101/2025.06.13.659490; this version posted June 15, 2025. The copyright holder for this preprint (which was not certified by peer review) is the author/funder, who has granted bioRxiv a license to display the preprint in perpetuity. It is made available under aCC-BY 4.0 International license.

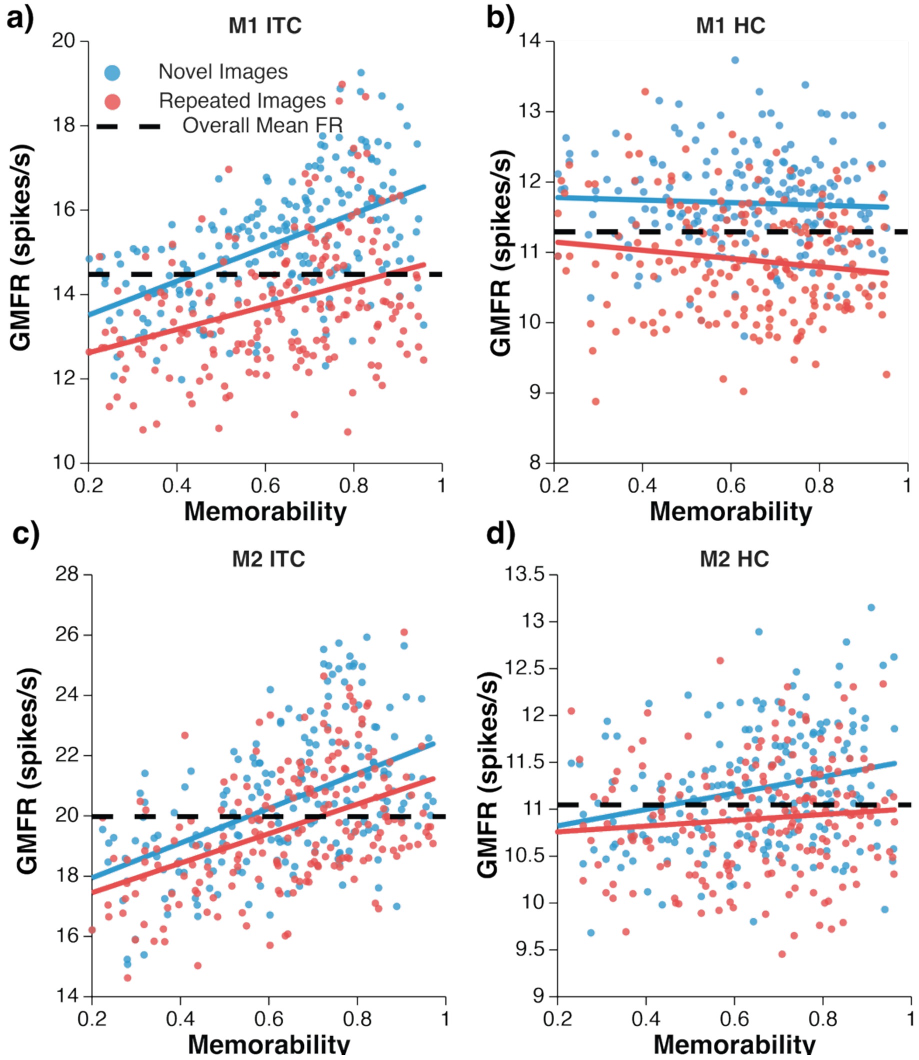

Figure S3: *Memorability modulation was attenuated between ITC and HC in both monkeys*. Same conventions as Fig. 2c-d. a) In monkey 1 ITC, mean RS was 9.0%. For novel images, Pearson's r(206) = .56, p = 6.5-e-19 and for repeated images r(206) = .37, p = 2.8e-8. b) In monkey 1's HC, mean RS was 6.6%. For novel images r(206) = -0.05, p = .48 and for repeated images r(206) = -0.14, p = .05. c) In monkey 2's ITC, mean RS was 4.0%. For novel images r(206) = .52, p = 8.6e-16, and for repeated images r(206) = .49, p = 5.2e-14. d) For monkey 2's HC, mean repetition suppression was 2.6% and for novel images r(206) = 3.43e-5 and for repeated images r(206) = .11, p = .13. Testing for attenuation of MB between ITC and HC in each monkey (see Methods): For monkey 1 novel ITC β=4.011, HC β=-0.183 (H0: Δβ = 0 p < .0001), for repeated ITC β=2.751, HC β=-0.588 (H0: Δβ = 0 p = .0002). For monkey 2, novel ITC β=5.766, HC β=0.874 (H0: Δβ = 0 p = 0.0247), for repeated ITC β=4.902, HC β=0.308 (H0: Δβ = 0 p = 0.0231).

bioRxiv preprint doi: https://doi.org/10.1101/2025.06.13.659490; this version posted June 15, 2025. The copyright holder for this preprint (which was not certified by peer review) is the author/funder, who has granted bioRxiv a license to display the preprint in perpetuity. It is made available under aCC-BY 4.0 International license.

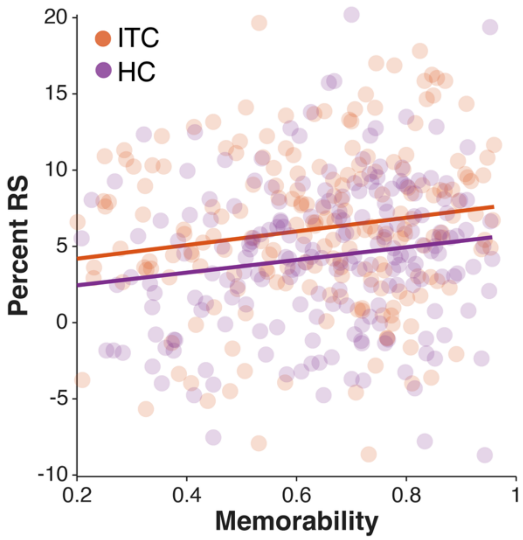

Fig. S4: *Repetition suppression (RS) modestly increases as a function of memorability in both ITC and HC.* Each dot represents the amount of RS evoked across all units by an image of a given memorability; lines are linear regressions. For ITC, $r(206) = 0.17$, $p = .014$. For HC, $r(206) = .17$, $p = 0.016$.

bioRxiv preprint doi: https://doi.org/10.1101/2025.06.13.659490; this version posted June 15, 2025. The copyright holder for this preprint (which was not certified by peer review) is the author/funder, who has granted bioRxiv a license to display the preprint in perpetuity. It is made available under aCC-BY 4.0 International license.

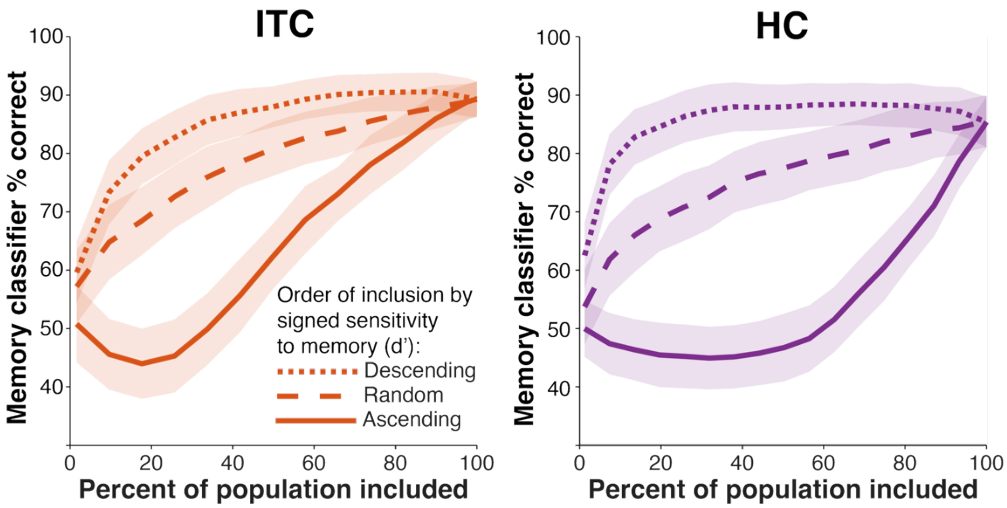

Figure S5: *Memory is reflected as repetition suppression in both ITC and HC.* To determine if the small fraction of RE units in Fig. 3a were noise or were contributing to memory, we performed a ranked d' analysis. Here, we trained a weighted linear classifier (the FLD classifier, see Methods) applied to neural data in each population. We ranked the units by their signed d' of memory (i.e., in the "descending" order of inclusion, units with the most repetition suppression received the highest ranks) and cumulatively included portions of the population, testing for memory performance of each subpopulation. Broadly, we see a similar pattern between ITC and HC. When including units starting with the lowest d' first (i.e., the repetition enhanced units; solid line), memory decoder performance in both brain areas did not exceed chance until well after repetition suppressed units were included in the population. Similarly, in both populations, a large amount of the classifier performance could be accounted for with a small number of the highly repetition suppressed units (dotted lines). For this classifier, the memorability alignment of the pseudopopulation was shuffled to minimize the effects of the 'paradox of memorability' (see Methods). Error shadows show the standard deviation of classifier performance across 200 cross-validations at each population size.

bioRxiv preprint doi: https://doi.org/10.1101/2025.06.13.659490; this version posted June 15, 2025. The copyright holder for this preprint (which was not certified by peer review) is the author/funder, who has granted bioRxiv a license to display the preprint in perpetuity. It is made available under aCC-BY 4.0 International license.

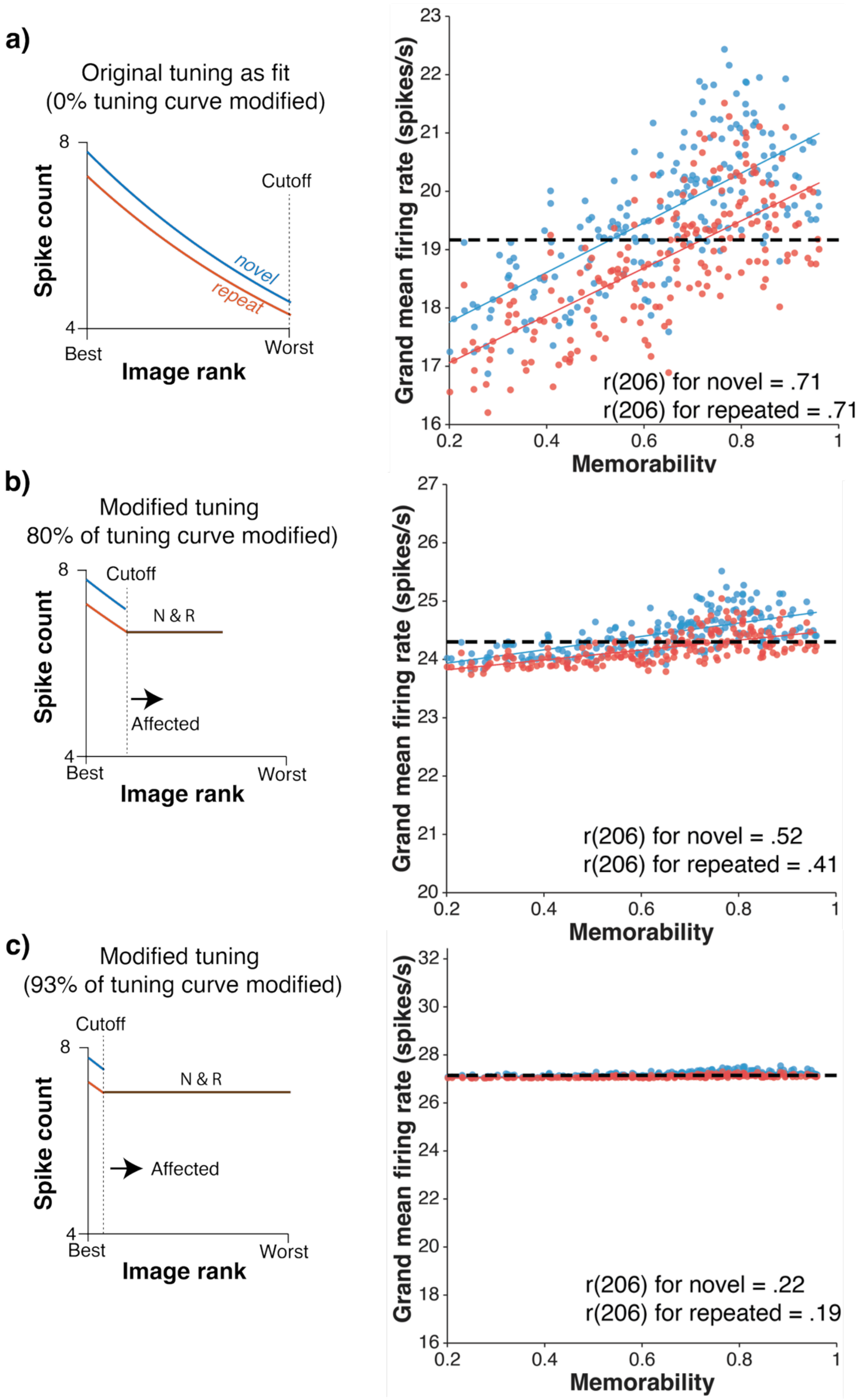

Figure S6 *Example synthetic ITC populations.* Example fit tuning curve (left) used to generate synthetic population. Equivalent analysis to Fig. 2c-d on synthesized data (right). The synthetic population was

bioRxiv preprint doi: https://doi.org/10.1101/2025.06.13.659490; this version posted June 15, 2025. The copyright holder for this preprint (which was not certified by peer review) is the author/funder, who has granted bioRxiv a license to display the preprint in perpetuity. It is made available under aCC-BY 4.0 International license.

generated by fitting exponential tuning curves to each unit and using a Poisson process to simulate spikes (see Methods for details). b-c) Populations generated based on modified tuning curves at 80% tuning thresholded (b) and 93% of tuning thresholded (c).
